# Erythropoietin treatment and osteoporotic fracture risk in hemodialysis patients: A nationwide population-based study

**DOI:** 10.1016/j.afos.2024.11.002

**Published:** 2024-12-02

**Authors:** Ching-Yu Lee, Fung-Chang Sung, Peir-Haur Hung, Chih-Hsin Muo, Meng-Huang Wu, Tsung-Jen Huang, Chih-Ching Yeh

**Affiliations:** a Department of Orthopedics, Taipei Medical University Hospital, Taipei, Taiwan; b Department of Orthopedics, School of Medicine, College of Medicine, Taipei Medical University, Taipei, Taiwan; c Orthopedics Research Center, Taipei Medical University Hospital, Taipei, Taiwan; d Department of Health Services Administration, China Medical University, Taichung, Taiwan; e Management Office for Health Data, China Medical University Hospital, Taichung, Taiwan; f Department of Food Nutrition and Health Biotechnology, Asia University, Taichung, Taiwan; g Department of Internal Medicine, Ditmanson Medical Foundation Chia-Yi Christian Hospital, Chiayi, Taiwan; h Department of Applied Life Science and Health, Chia-Nan University of Pharmacy and Science, Tainan, Taiwan; i Graduate Institute of Clinical Medical Science, College of Medicine, China Medical University, Taichung, Taiwan; j School of Public Health, College of Public Health, Taipei Medical University, New Taipei, Taiwan; k Master Program in Applied Epidemiology, College of Public Health, Taipei Medical University, New Taipei, Taiwan; l Department of Public Health, College of Public Health, China Medical University, Taichung, Taiwan; m Cancer Center, Wan Fang Hospital, Taipei Medical University, Taipei, Taiwan

**Keywords:** Erythropoietin, End-stage renal disease, Hemodialysis, Osteoporotic fracture risk

## Abstract

**Objectives:**

Concerns about erythropoietin (EPO) therapy for anemia in patients with end-stage renal disease (ESRD) contributing to potential bone loss and increased fracture risks are growing. This study investigated the impact of EPO administration on the risk of common osteoporotic fractures in ESRD patients.

**Methods:**

This population-based retrospective cohort study compared EPO users and non-EPO users among ESRD patients undergoing hemodialysis, diagnosed with ESRD between 2000 and 2014 identified from the National Health Insurance Research Database of Taiwan. The cohorts were matched at a propensity score ratio of 1:1, resulting in equal sample sizes of 2839. Variables related to comorbidities were considered.

**Results:**

EPO users exhibited higher cumulative incidences of major osteoporotic fractures, hip fractures, spine fractures, and wrist fractures compared with the non-EPO user (all P < 0.001). In adjusted Cox regression models, higher adjusted subdistribution hazard ratios (aSHRs) were observed for major osteoporotic fractures (2.41, 95% confidence interval [CI] = 2.01–2.89), osteoporotic hip fractures (2.19, 95% CI = 1.69–2.85), spine fractures (2.50, 95% CI = 1.87–3.34), and wrist fractures (2.34, 95% CI = 1.44–3.78) in EPO users than in non-EPO users. The risk of major osteoporotic fractures significantly increased with increasing EPO doses (P for trend < 0.0001), and a similar trend was observed for the risks of osteoporotic spine and wrist fractures.

**Conclusions:**

Our findings suggest that EPO treatment in patients with ESRD undergoing hemodialysis is associated with an increased risk of osteoporotic fractures.

## 1 Introduction

Osteoporosis, which is characterized by the loss of bone mass, compromised skeletal microarchitecture, and increased susceptibility to fractures, poses a substantial global health challenge [1]. Osteoporotic fractures or fragility fractures are caused by minimal trauma (low-energy trauma), such as a fall from standing height or less [2]. Predominantly occurring in the hips (proximal femur), spine (vertebral body), and wrists (distal radius) [3], these fractures significantly contribute to morbidity and mortality and impose a substantial socioeconomic burden [2]. End-stage renal disease (ESRD) is a substantial public health concern, and osteoporosis is prevalent among patients with ESRD. This prevalence is attributed to the abnormal metabolism of vitamin D and calcium, resulting in disturbances in mineralization and a decline in bone density and quality [4]. The intersection of ESRD and osteoporosis in these patients increases the challenges for their treatment, indicating the importance of comprehensive care strategies for simultaneously addressing both conditions.

To address anemia, a common complication of ESRD, erythropoietin (EPO) has emerged as a fundamental treatment. As a pivotal hormone regulating red blood cell production, EPO has revolutionized the treatment of anemia in patients with ESRD, leading to improved hemoglobin levels and alleviation of symptoms such as fatigue and weakness [5]. Despite commendable advances in anemia treatment, concerns regarding the potential association between EPO administration and an elevated risk of bone loss or fractures are growing [[6], [7], [8], [9]]. In vivo experiments have revealed the deleterious effects of EPO on bone mass, as evidenced by the trabecular bone loss observed in transgenic mice overexpressing EPO and in mice treated with exogenous EPO [10,11]. Clinical studies have further supported an association between EPO and the increased risk of fractures [8,9]. The population-based Osteoporotic Fractures in Men (MrOS) study conducted in Sweden discovered a correlation between elevated plasma EPO levels and the elevated risk of fractures [8]. Additionally, an analysis of the United States Renal Data System dataset revealed a proportional variation in hip fracture incidence with the annual sum of EPO doses in patients undergoing hemodialysis [9]. Consequently, the dual roles of EPO, serving as a crucial treatment for anemia and potentially contributing to bone fragility, necessitates a thorough exploration in patients with ESRD undergoing hemodialysis.

Currently, no additional evidence supporting an association between EPO treatment and the increased risk of osteoporotic fractures in patients with ESRD is available. Non-hip fractures attributable to osteoporosis constitute a substantial portion of overall osteoporotic fractures, with consequences comparable to the adverse effects of hip fractures [12]. Studies specifically examining non-hip fractures associated with EPO treatment are currently lacking. The primary objective of the present population-based study is to explore the potential adverse effects of EPO treatment on the risk of common osteoporotic fractures, including hip and other fractures, in patients with ESRD using insurance claims data of Taiwan.

## 2 Methods

### 2.1 *Data sources*

Our retrospective cohort study employed claims data extracted from the National Health Insurance Research Database (NHIRD), accessible at the Health and Welfare Data Science Center (HWDSC) in Taiwan. Since March 1, 1995, Taiwan has implemented a single-payer system, the National Health Insurance (NHI) program, covering > 99% of the country's citizens. The NHIRD contains information including beneficiary details, inpatient and outpatient claims, and data on catastrophic illnesses. To ensure patient privacy, the data were linked using decoded personal identification numbers. The *International Classification of Diseases,* 9th Revision, Clinical Modification (*ICD-*9-CM) was used for disease identification until 2016, and from 2016, the *ICD-*10-CM was used within the databases. The WHO Anatomical Therapeutic Chemical Classification System (ATC codes) was employed for classifying medication usage. The study's research proposal was approved by the Research Ethics Committee of China Medical University and Hospital in Taiwan (CRREC-107-021).

### 2.2 *Study cohorts*

**Fig. 1:**
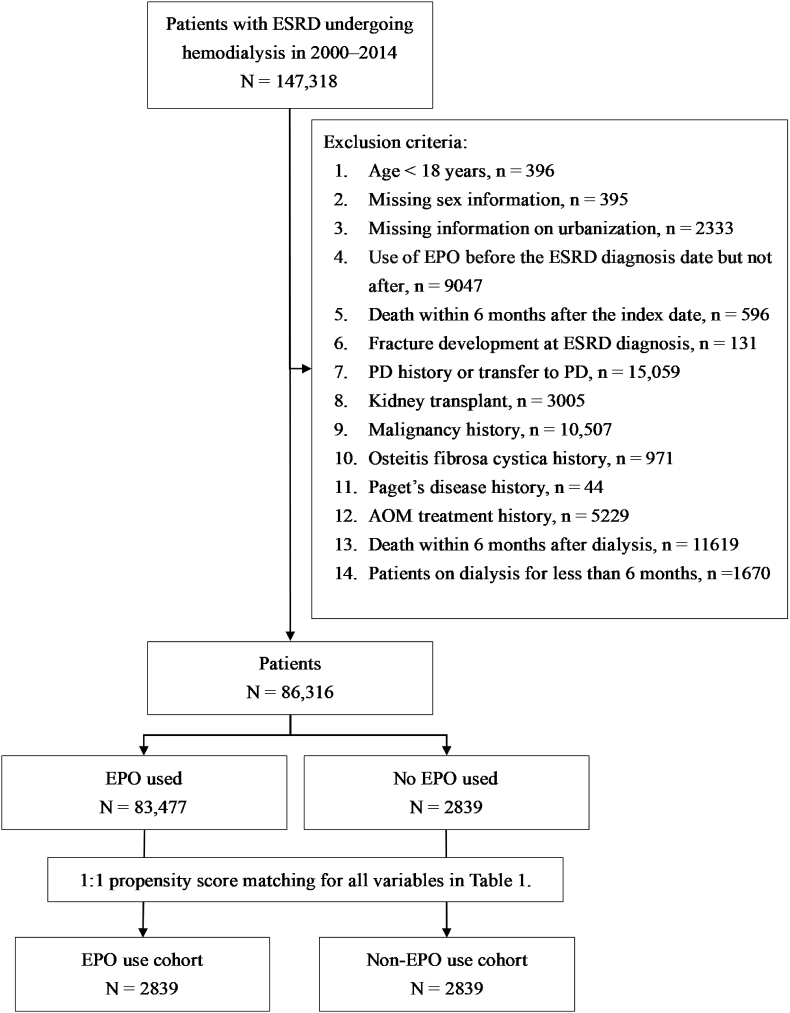
Flowchart of study cohort selection. ESRD, end-stage renal disease; PD, peritoneal dialysis; AOM, anti-osteoporotic medicine; EPO, erythropoietin.

A total of 147,318 patients with newly diagnosed ESRD who underwent hemodialysis between 2000 and 2014 were identified from the NHIRD at the HWDSC. Patients with a history of peritoneal dialysis, those undergoing anti-osteoporotic medication treatment, those who were kidney transplant recipients, and those with comorbidities posing risks for pathologic fractures (eg, malignancy, osteitis fibrosa cystica, and Paget's disease) were excluded from the analysis. Additionally, individuals younger than 18 years, those on dialysis for less than 6 months, those using EPO before the ESRD diagnosis date, individuals deceased within 6 months after the index date, or those with missing information on the residential area were also excluded. Following the application of these criteria, a cohort of 86,316 patients with ESRD remained. Among them, 83,477 patients had received EPO treatment, whereas 2839 patients had not. The index date was defined as the date of initiation of EPO therapy in patients with ESRD. Propensity scores were calculated for each individual through logistic regression, with adjustments for age, sex, ESRD diagnosis date, urbanization level, and comorbidities. By employing greedy algorithms, the cohort of non-EPO users and the propensity-score-matched comparison cohort of EPO users were established, ensuring equal sizes in each group, as illustrated in Fig. 1.

### 2.3 *Demographics, comorbidity, EPO exposure, and outcome*

The claims data comprise demographic information on age, gender, and urbanization level. Seven levels of urbanization were identified: Levels 1 and 2 were classified as “urban”, levels 3 and 4 as “suburban”, and levels 5–7 as “rural” [13]. Besides the common comorbidities associated with osteoporosis like hypertension, hyperlipidemia, diabetes mellitus, coronary heart disease, congestive heart failure, atrial fibrillation, stroke, anemia, osteoporosis, and a history of fractures [[14], [15], [16], [17], [18], [19], [20]], additional factors contributing to osteoporotic fractures including conditions predisposing to falls, and comorbidities leading to osteoporosis were also considered [21,22]. Therefore, we incorporated risk factors for falls, such as arthropathy, alcoholism, dizziness, and visual impairment [23,24]. Additionally, we considered risk factors for osteoporosis, including endocrine disorders, malnutrition, chronic obstructive pulmonary disease (COPD), chronic liver disease, alcoholic and viral hepatitis [21,23]. Each comorbidity was identified 1 year prior to the date of ESRD. The EPO dosage per week (expressed as the daily defined dose [DDD]) was estimated by dividing the total EPO usage during the study period by the sum of the follow-up weeks. We stratified the EPO dosage into four levels by quartiles (Q1 level: 1–1108 DDDs, Q2 level: 1109–3335 DDDs, Q3 level: 3336–11,762 DDDs, and Q4 level: > 11,763 DDDs). The follow-up person-years were recorded from the index date to the occurrence of osteoporotic fractures, death, or the end of 2016 for both cohorts. The three most prevalent osteoporotic fractures were as follows: hip fractures (*ICD-9* codes: 733.14, 820.0, and 820.2, *ICD-10* codes: S72.0, S72.1, and S72.2, or surgical procedure codes: 64029B, 64041C, and 64170B), spine fractures (*ICD-9* codes: 733.13, 805.0, 805.2, 805.4, 805.8, 806.0, 806.2, 806.4, and 806.8, *ICD-10* codes: M48.4, M48.5, S12, S22.0, S32.0, and S32.1, or surgical procedure codes: 33126B, 33127B, 64042C, and 64160B), and distal radial fractures (*ICD-9* codes: 733.12 and 813.4, *ICD-10* codes: S52.5, or surgical procedure codes: 64032B, 64044C, and 64271C). Major osteoporotic fractures were defined as fractures of the hip, spine, wrist, and proximal humerus [3,25]. Humerus fracture codes included *ICD-9* codes 812.0 and 733.11, *ICD-10* code S42.2, or surgical procedure codes 64239B and 64043C. Because osteoporotic fractures result from low-energy trauma, fractures associated with concomitant motor vehicle accidents or high-impact trauma recorded on the same day were excluded from the analysis.

### 2.4 *Statistical analysis*

A comparative analysis of EPO and non-EPO user cohorts was conducted, and the results are presented as numbers and percentages. A standardized difference of > 0.1 was considered significant, indicating a notable difference in the balance of variables between the two cohorts. We computed the incidence of osteoporotic fractures by dividing the number of people diagnosed with osteoporotic fractures by the follow-up time (person-years) for both cohorts. The incidence rates were estimated for major osteoporotic fractures, and the osteoporotic hip fractures, osteoporotic spine fractures and osteoporotic wrist fractures. We further evaluated incidence rates in the EPO cohort by the quartile level of EPO prescribed for patients. The adjusted subdistribution hazard ratio (aSHR) was calculated for the EPO cohort compared to the non-EPO cohort. This calculation involved adjustments for sex, age, and all comorbidities using a multivariable Cox proportional hazards regression model with time-dependent covariates. Additionally, considering the higher mortality in ESRD patients, the analysis accounted for death as a competing risk through Cox proportional hazards regression based on the Fine and Gray model [26,27].

The linear trend test was utilized to determine the prospective trends for observation of the dose-response relationship between EPO dosage and the risk of osteoporotic fractures. The risks associated age and sex were also considered and estimated. The cumulative incidence rates were estimated and plotted by Kaplan-Meier curves considering the competing risk of death. The data was analyzed with SAS 9.4 software (SAS Institute Inc., Cary, NC, USA). A P-value of less than 0.05 was determined to be statistically significant.

## 3 Results

**Fig. 2:**
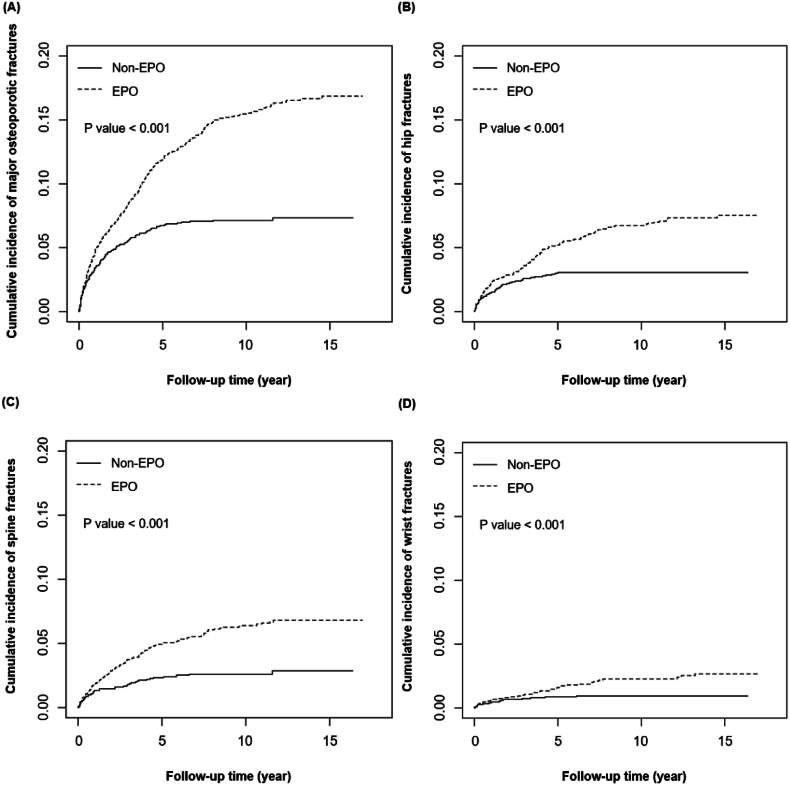
Cumulative incidence of (A) major osteoporotic fracture, (B) osteoporotic hip fracture, (C) osteoporotic spine fracture, and (D) osteoporotic wrist fracture in two EPO cohorts, considering death as a competing risk.

Following propensity score matching, the study cohorts comprised 2839 individuals of none EPO users and an equivalent number of EPO users. Both cohorts exhibited comparable distributions for all baseline variables, as outlined in Table 1. In each cohort, over 80% of the ESRD patients were aged over 55, and more than half of them resided in urban areas. The predominant comorbidities between both cohorts were hypertension, diabetes, visual disorders, anemia, and coronary heart diseases. In Fig. 2, the cumulative incidence rates of the major osteoporotic fractures, hip fractures, spine fractures, and wrist fractures was higher in EPO cohort than in the non-EPO cohort (all P < 0.001).

**Table 1:** Baseline demographic characteristics and comorbidities between EPO users and non-EPO users.

Variable	Total		Non-EPO users		EPO users		P	Standardized difference
	N = 5678		N = 2839		N = 2839			
	N	%	N	%	N	%		
Age, yrs							0.516	
18–44	165	2.91	80	2.82	85	2.99		0.010
45–54	489	8.61	258	9.09	231	8.14		0.034
55–64	951	16.8	481	16.9	470	16.6		0.010
65–74	1440	25.4	731	25.8	709	25.0		0.018
≥ 75	2633	46.4	1289	45.4	1344	47.3		0.039
Sex							0.770	
Male	3083	54.3	1536	54.1	1547	54.5		0.008
Female	2595	45.7	1303	45.9	1292	45.5		0.008
Urbanization							0.958	
Urban	3107	54.7	1559	54.9	1548	54.5		0.008
Suburban	1882	33.2	937	33.0	945	33.3		0.006
Rural	689	12.1	343	12.1	346	12.2		0.003
**General comorbidity**								
Hypertension	5385	94.8	2698	95.0	2687	94.7	0.509	0.018
Hyperlipidemia	2680	47.2	1334	45.0	1346	47.4	0.750	0.008
Diabetes	4262	75.1	2119	74.6	2143	75.5	0.462	0.020
Coronary heart disease	3312	58.3	1657	58.4	1655	58.3	0.957	0.001
Congestive heart failure	2748	48.4	1380	48.6	1368	48.2	0.750	0.008
Atrial fibrillation	486	8.56	242	8.52	244	8.59	0.924	0.003
Stroke	2517	44.3	1278	45.0	1239	43.6	0.298	0.028
Anemia	3377	59.5	1704	60.0	1673	58.9	0.402	0.022
Osteoporosis	494	8.70	241	8.49	253	8.91	0.572	0.015
Fracture history	1684	29.7	836	29.5	848	29.9	0.727	0.009
**Risk factors for falls**								
Arthropathies	2489	43.8	1242	43.8	1247	43.9	0.894	0.004
Alcoholism and dizziness	1018	17.9	516	18.2	502	17.7	0.628	0.013
Visual disorders	3612	63.6	1809	63.7	1803	63.5	0.869	0.004
**Risk factors for osteoporosis**								
Endocrine disorders and malnutrition	562	9.90	276	9.72	286	10.1	0.657	0.012
Chronic obstructive pulmonary disease	1707	30.1	831	29.3	876	30.9	0.193	0.035
Chronic hepatic disease and hepatitis	2070	36.5	1029	36.3	1041	36.7	0.741	0.009
EPO (DDD/week), median (IQR)	3336.4	(10643.9)			3336.4	(10643.9)		
Follow-up years, mean (SD)	2.88	(3.07)	1.52	(1.75)	4.23	(3.49)	<0.0001	0.98

EPO, erythropoietin; DDD, daily defined dose; IQR, interquartile range; SD, standard deviation.

Table 2 shows that the overall incidence rate of each type of fracture was higher in the EPO cohort than in the non-EPO cohort. The estimated aSHR of the major osteoporotic fractures was 2.41 (95% CI = 2.01–2.89) for EPO users. Similarly, increased risk of osteoporotic fractures of the hip (aSHR = 2.19, 95% CI = 1.69–2.85), spine (aSHR = 2.50, 95% CI = 1.87–3.34), and wrist (aSHR = 2.34, 95% CI = 1.44–3.78) were observed in EPO users. The aSHR increased significantly with increasing EPO dose for major osteoporotic, spine and wrist fractures (P for trend < 0.0001), despite reduced hazards appeared at Q3 dose of EPO.

**Table 2:** Incidence rate and EPO cohort to non-EPO cohort hazard ratio of fractures by fracture type and quartile dosage of EPO.

Fracture	EPO (DDD/week)	N	Event	PY	Rate^a^	aSHR (95% CI)^b^
Major osteoporotic fracture	No	2839	165	4352	37.92	Ref.
	Yes	2839	395	12,096	32.65	2.41 (2.01–2.89)
	Q1	703	91	2674	34.03	2.23 (1.73–2.88)
	Q2	716	105	2718	38.632	2.53 (1.99–3.21)
	Q3	711	94	3439	27.33	2.26 (1.76–2.91)
	Q4	709	105	3265	32.16	2.62 (2.05–3.35)
	P for trend					< 0.0001
Osteoporotic hip fracture	No	2839	80	4352	18.38	Ref.
	Yes	2839	177	12,096	14.63	2.19 (1.69–2.85)
	Q1	703	42	2674	15.71	2.09 (1.44–3.05)
	Q2	716	49	2718	18.03	2.40 (1.70–3.40)
	Q3	711	46	3439	13.38	2.24 (1.57–3.21)
	Q4	709	40	3265	12.25	2.02 (1.38–2.96)
	P for trend					< 0.0001
Osteoporotic spine fracture	No	2839	65	4352	14.94	Ref.
	Yes	2839	164	12,096	13.56	2.50 (1.87–3.34)
	Q1	703	33	2674	12.34	2.02 (1.32–3.09)
	Q2	716	43	2718	15.82	2.59 (1.76–3.81)
	Q3	711	41	3439	11.92	2.46 (1.67–3.62)
	Q4	709	47	3265	14.40	2.93 (2.01–4.27)
	P for trend					< 0.0001
Osteoporotic wrist fracture	No	2839	23	4352	5.29	Ref.
	Yes	2839	55	12,096	4.55	2.34 (1.44–3.78)
	Q1	703	16	2674	5.98	2.74 (1.48–5.09)
	Q2	716	13	2718	4.78	2.19 (1.11–4.31)
	Q3	711	7	3439	2.04	1.17 (0.50–2.72)
	Q4	709	19	3265	5.82	3.31 (1.79–6.09)
	P for trend					0.002

CI, confidence interval; DDD, defined daily dose; EPO, erythropoietin; HR, hazard ratio; PY, person-years; SHR, subdistribution hazard ratio.

a Incidence rate, per 1000 person-years.

b Cox model adjusted for age, sex, urbanization level, and all comorbidities, with death as the competing factor.

Table 3 reveals a significantly higher incidence of major osteoporotic fractures in EPO users than in non-EPO users, regardless of age, with an aSHR of 1.88 (95% CI = 1.36–2.62) for individuals aged < 65 years and 2.63 (95% CI = 2.12–3.27) for those aged ≥ 65 years. Similarly, EPO users exhibited significantly higher rates of osteoporotic hip and spine fractures than their non-EPO counterparts, independent of age. Moreover, the risk of major osteoporotic fractures increased with the EPO dose, with the highest aSHR observed in the fourth quartile across both age groups (P for trend = 0.0002 and P for trend < 0.0001, respectively). No significant association was noted between EPO use and the risk of osteoporotic wrist fractures in individuals aged < 65 years. Conversely, in patients aged > 65 years, the risk of osteoporotic wrist fractures increased with increasing EPO doses (P for trend = 0.0009).

**Table 3:** Incidence rate and EPO cohort to non-EPO cohort hazard ratio of fractures by fracture type, quartile dosage of EPO and age.

Fracture	EPO (DDD/week)			< 65 years			65+ years
		Event	Rate^a^	aSHR (95% CI)^b^	Event	Rate^a^	aSHR (95% CI)^b^
Major osteoporotic fracture	No	53	24.06	Ref.	112	52.11	Ref.
	Yes	99	16.28	1.88 (1.36–2.62)	296	49.20	2.63 (2.12–3.27)
	Q1	19	15.54	1.54 (0.92–2.58)	72	49.61	2.50 (1.86–3.35)
	Q2	25	19.45	1.97 (1.24–3.13)	80	55.84	2.72 (2.05–3.62)
	Q3	30	15.85	2.07 (1.33–3.22)	64	41.39	2.37 (1.75–3.21)
	Q4	25	14.89	1.93 (1.20–3.12)	80	50.46	2.94 (2.21–3.92)
	P for trend			0.0002			< 0.0001
Osteoporotic hip fracture	No	20	9.08	Ref.	60	27.92	Ref.
	Yes	42	6.91	2.10 (1.24–3.56)	135	22.44	2.20 (1.62–2.98)
	Q1	8	6.54	1.70 (0.75–3.86)	34	23.43	2.16 (1.42–3.30)
	Q2	11	9.33	2.48 (1.22–5.04)	37	25.82	2.31 (1.54–3.46)
	Q3	11	5.81	1.99 (0.96–4.11)	35	22.63	2.37 (1.56–3.60)
	Q4	11	6.55	2.22 (1.06–4.64)	29	18.29	1.95 (1.25–3.03)
	P for trend			0.007			<0.0001
Osteoporotic spine fracture	No	22	9.99	Ref.	43	20.01	
	Yes	38	6.25	1.72 (1.02–2.90)	126	20.94	Ref.
	Q1	5	4.09	0.97 (0.37–2.54)	28	19.29	2.86 (2.02–4.06)
	Q2	9	7.00	1.68 (0.77–3.67)	34	23.73	2.48 (1.53–4.02)
	Q3	15	7.93	2.44 (1.28–4.66)	26	16.81	2.96 (1.88–4.65)
	Q4	9	5.36	1.65 (0.76–3.58)	38	23.97	2.45 (1.51–3.99)
	P for trend			0.014			3.56 (2.29–5.51)
Osteoporotic wrist fracture	No	12	5.45	Ref.	11	5.12	< 0.0001
	Yes	19	3.12	0.64 (0.32–1.29)	36	5.98	Ref.
	Q1	6	4.91	2.10 (0.84–5.24)	10	6.89	3.16 (1.61–6.21)
	Q2	4	3.11	1.35 (0.44–4.17)	9	6.28	3.42 (1.46–8.02)
	Q3	4	2.11	1.19 (0.38–3.67)	3	1.94	3.03 (1.26–7.30)
	Q4	5	2.98	1.66 (0.59–4.70)	14	8.83	1.09 (0.30–3.91)
	P for trend			0.434			5.07 (2.29–11.2)

CI, confidence interval; DDD, defined daily dose; EPO, erythropoietin; HR, hazard ratio; PY, person-years; SHR, subdistribution hazard ratio.

a Incidence rate, per 1000 person-years.

b Cox model adjusted for age, sex, urbanization level, and all comorbidities, with death as the competing factor.

The results of the assessment of the risk of osteoporotic fractures within the hemodialysis patient cohort using EPO, stratified by sex, are presented in Table 4. Regardless of sex, EPO users exhibited significantly higher risks of osteoporotic hip, spine, and major osteoporotic fractures than non-EPO users. However, the risk of osteoporotic wrist fractures was significantly different between EPO users and non-EPO users in women (aSHR = 2.99, 95% CI = 1.55–5.40), but not in men. Apart from osteoporotic all fracture types in men, the risks of osteoporotic major, spine and wrist fractures increased with the EPO dose, with the highest aSHR observed in the fourth quartile in female.

**Table 4:** Incidence rate and EPO cohort to non-EPO cohort hazard ratio of fractures by fracture type, quartile dosage of EPO and sex.

Fracture	EPO (DDD/week)			Men			Women
		Event	Rate^a^	aSHR (95% CI)^b^	Event	Rate^a^	aSHR (95% CI)^b^
Major osteoporotic fracture	No	66	26.95	Ref.	99	52.03	Ref.
	Yes	158	23.41	2.35 (1.77–3.13)	237	44.31	2.48 (1.97–3.12)
	Q1	34	21.84	2.01 (1.32–3.04)	57	51.01	2.43 (1.77–3.34)
	Q2	40	25.84	2.28 (1.56–3.34)	65	55.54	2.79 (2.05–3.79)
	Q3	45	24.42	2.71 (1.85–3.96)	49	30.69	1.96 (1.40–2.74)
	Q4	39	21.66	2.42 (1.63–3.58)	66	45.008	2.77 (2.03–3.77)
	P for trend			< 0.0001			< 0.0001
Osteoporotic hip fracture	No	33	13.48	Ref.	47	24.70	Ref.
	Yes	83	12.30	2.44 (1.64–3.64)	94	17.58	2.02 (1.43–2.86)
	Q1	16	10.28	1.87 (1.03–3.40)	26	23.27	2.29 (1.41–3.70)
	Q2	25	16.15	2.82 (1.71–4.66)	24	20.51	2.13 (1.31–3.45)
	Q3	23	12.48	2.74 (1.61–4.67)	23	14.41	1.89 (1.16–3.09)
	Q4	19	10.55	2.33 (1.33–4.08)	21	14.34	1.80 (1.08–3.00)
	P for trend			< 0.0001			0.002
Osteoporotic spine fracture	No	25	10.21	Ref.	40	21.02	Ref.
	Yes	62	9.19	2.40 (1.51–3.83)	102	19.07	2.58 (1.79–3.72)
	Q1	15	9.64	2.31 (1.22–4.39)	18	16.11	1.86 (1.07–3.26)
	Q2	11	7.11	1.64 (0.81–3.32)	32	27.34	3.33 (2.10–5.28)
	Q3	21	11.40	3.29 (1.85–5.86)	20	12.53	1.93 (1.14–3.27)
	Q4	15	8.33	2.41 (1.27–4.59)	32	21.86	3.24 (2.04–5.16)
	P for trend			0.0001			<0.0001
Osteoporotic wrist fracture	No	9	3.68	Ref.	14	7.36	Ref.
	Yes	13	1.93	1.40 (0.60–3.27)	42	7.85	2.99 (1.65–5.40)
	Q1	3	1.93	1.28 (0.35–4.74)	13	11.63	3.80 (1.85–7.82)
	Q2	4	2.58	1.66 (0.51–5.37)	9	7.69	2.62 (1.14–6.05)
	Q3	1	0.54	0.44 (0.06–3.45)	6	3.76	1.61 (0.62–4.18)
	Q4	5	2.78	2.22 (0.74–6.63)	14	9.56	4.02 (1.91–8.45)
	P for trend			0.378			0.002

CI, confidence interval; DDD, defined daily dose; EPO, erythropoietin; HR, hazard ratio; PY, person-years; SHR, subdistribution hazard ratio.

a Incidence rate, per 1000 person-years.

b Cox model adjusted for age, sex, urbanization level, and all comorbidities, with death as the competing factor.

## 4 Discussion

Data on the potential association between EPO therapy and risk of osteoporotic fractures in patients with ESRD undergoing hemodialysis are limited. Osteoporotic fractures are caused by mechanical forces that would not typically cause fractures, which are termed low-energy trauma, such as a fall from standing height or less. The most prevalent osteoporotic fractures involve the hips, spine, and wrists. According to the Fracture Risk Assessment Tool (FRAX) for individuals with osteoporosis, major osteoporotic fractures include fractures involving the spine, hip, wrist, and shoulder [3,25]. To the best of our knowledge, this study is the first to demonstrate a significant association between EPO treatment and the risk of osteoporotic fractures in patients with ESRD by using real-world data. Among patients with ESRD undergoing hemodialysis, EPO treatment increased the risk of not only major osteoporotic fractures but also osteoporotic fractures affecting the hip, spine, and wrist.

In the present study, the aSHRs of osteoporotic fractures for the EPO cohort were all near 2.0 or higher, compared with non-EPO cohort, indicating the osteoporotic fracture hazards appeared mainly in survived patients on the dialysis with EPO therapy. The aSHRs showed a dose-response relationship between osteoporotic fracture hazards and DDD levels of EPO. The cumulative incidence rates of osteoporotic fractures were all higher in EPO users than non-EPO users measured with the competing risk of death considered. Similarly, another research utilizing the US Renal Data System observed a significant increase in hip fracture incidences among hemodialysis patients with dose response relationship of EPO [9]. However, the US study failed explicitly to state whether EPO increases the risk of “osteoporotic” hip fractures. It is likely that the measured hip fractures included not only “osteoporotic” hip fractures but also high-energy traumatic fractures or other pathologic fractures linking to malignancy, osteitis fibrosa cystica, and Paget's disease.

Both the US study and our study suggest that exogenous EPO administration does indeed contribute to a heightened incidence of fractures among hemodialysis patients. Moreover, the Sweden MrOS study in 2020 has explored the association between fracture incidence and endogenous EPO levels in elderly men with normal renal function over a 10-year follow-up [13]. The results demonstrated that men with a higher serum level of endogenous EPO were at an elevate risk of incident fractures, including major osteoporotic fractures and clinical vertebral fractures. Hence, based on findings in these three studies, we could expect the EPO therapy may indeed raise the likelihood of osteoporotic fracture development, with a relationship dependent on dosage.

Both in vitro and in vivo studies have explored the impact of EPO on bone homeostasis; however, the impact of EPO on bone remains a topic of debate [6]. Recent research has demonstrated the presence of the EPO receptor (EPO-R) in bone cells, such as osteoclasts and osteoblasts [6]. Osteoclasts, derived from the monocyte lineage, play a role in bone resorption, whereas osteoblasts, originating from mesenchymal stem cells, are responsible for bone formation. In vitro studies have indicated that EPO can directly stimulate osteoclastogenesis by activating EPO receptors in osteoclast precursors [7,10]. Hiram-Bab and her colleagues observed an increase in osteoclastogenesis and a reduction in bone mineral density in a mouse model characterized by EPO overproduction compared with wild-type mice [10]. Moreover, they observed that EPO administration stimulated bone resorption, reduced bone formation, and induced osteoclastogenesis, leading to bone loss in adult mice treated with EPO injection compared with adult mice treated with saline injection [10]. Conversely, EPO has been demonstrated to promote the proliferation and differentiation of osteoblasts in human osteoblast cell lines and mouse osteoblasts [[28], [29], [30], [31]]. Additionally, in vivo EPO treatment has been reported to confer beneficial effects on fracture healing, such as improved callus formation and increased mineral content, in animal fracture models [[32], [33], [34], [35]], and this treatment exhibits osteogenic capacity in newborn mice [36].

Some studies have attributed variations in the effects of EPO on bone metabolism observed in both in vitro and in vivo settings to environmental conditions and age [6,10]. Models simulating bone fracture healing or the regeneration of newborn bones present distinct environmental conditions, including inflammatory reactions, hypoxia, and neoangiogenesis [37]. However, Orth and colleagues explored the in vivo association between EPO and aging in the context of fracture healing and discovered that EPO did not improve fracture healing in aged mice. Instead, administering EPO resulted in heightened bone fragility [38]. An animal study has shown that EPO treatment could induce bone loss in mice [11]. The EPO-induced decreases in trabecular bone mineral density in the mouse femurs and vertebrae were significantly irreversible after two weeks of EPO administration and then six weeks of drug withdrawal.

In summary, the complexity of EPO functions in bone cells under various environmental conditions is highlighted by both in vitro and in vivo studies. EPO may have adverse effects on bone mass in adults or elderly people. These preclinical and clinical findings suggest that EPO-induced bone loss may be dose-dependent and irreversible. Therefore, considering that patients requiring EPO treatment are often susceptible to osteoporosis, it is crucial to administer the lowest effective EPO dose to mitigate the adverse consequences of osteoporosis exacerbation.

This study employed a large-scale population-based data survey to establish a propensity-matched cohort for long-term follow-up, and this study investigated the impact of EPO treatment on the increased risk of osteoporotic fractures in patients with ESRD receiving hemodialysis. However, several limitations should be considered. One limitation concerns the identification of diseases using *ICD-9* codes from claims data before 2016, which may raise concerns about diagnostic accuracy. Nevertheless, the NHI Administration of Taiwan routinely conducts cross-checks of diagnoses, and the validity of claims data for ESRD and osteoporotic fractures has been affirmed in other studies [[39], [40], [41], [42]]. Another concern is the potential confounding of indication for ESRD patients requiring erythropoietin. Therefore, we used propensity score matching to establish our study cohorts, which could control for all relevant confounding indicators associated with anemia, osteoporosis, and the history of fracture. Additionally, the NHIRD lacks quantitative data from hematologic, biochemical, and imaging examinations, such as hemoglobin levels, 25-hydroxy vitamin D levels, and bone mineral density, which may affect the incidence of osteoporosis. Although the influence of likely confounding from the above-mentioned factors could not be ruled out, we used multivariate Cox models to compute aSHRs, accounting for age, sex, urbanization, and various comorbidities, including endocrine disorders and COPD, to mitigate potential residual confounding effects. Endocrine disorders include vitamin D deficiency, and COPD diagnosis not only is considered as a proxy measure for smoking [43] but also recognized as a risk factor for osteoporosis. Given the high mortality rate among ESRD patients on hemodialysis, we calculated hazard ratios and the cumulative incidence of osteoporotic fractures while accounting for the influence of death as a competing risk. The sample size in this study was relatively modest compared to other health insurance database studies. This is primarily due to stringent inclusion criteria for osteoporotic fractures which excluded patients linked to high-impact trauma, malignancy, osteitis fibrosa cystica, and Paget's disease.

In examining the relationship between EPO dosage and fracture risk, we found that while the aSHRs across quartiles do not show a strictly linear increase, the highest quartile (Q4) generally presents higher hazards than lower quartiles (Q1), particularly for major osteoporotic, spine, and wrist fractures. This observation aligns with the notion of increased fracture risk at higher cumulative doses of EPO among survivals, even though there are individual fluctuations in aSHR measures. For osteoporotic hip fractures, however, we observed lower incidence rates in Q3 and Q4 compared to Q1 and Q2. This pattern likely reflects fracture-specific characteristics, as hip fractures are more closely related to fall risk and mobility issues rather than solely bone density or quality. Patients in higher dosage quartiles (Q3 and Q4) might have received more intensive clinical management, including fall prevention measures, which could have mitigated hip fracture risk despite higher EPO exposure. Thus, while variability exists for specific fracture types, the overall trend remains evident. Lastly, serum ferritin and transferrin saturation (TSAT) levels used to adjust EPO therapy in patients undergoing hemodialysis were not available in our data. Therefore, the accurate administration of iron could not be assessed. However, the NHI mandates maintaining serum ferritin levels > 100 ng/mL and/or TSAT > 20% for patients receiving EPO therapy, suggesting alignment between our study's baseline iron parameters and the insurance reimbursement criteria for all EPO users.

## 5 Conclusions

Patients with ESRD undergoing hemodialysis receiving EPO treatment may have an elevated risk of osteoporotic fractures. The administration of EPO was associated not only with an increased risk of major osteoporotic fractures but also with the risks of osteoporotic fractures specifically affecting the hip, spine, and wrist. A notable dose–response relationship was observed, and an increase in the EPO dosage was correlated with an increased risk of osteoporotic fractures.

## Data availability statement

The Taiwan National Health Insurance database was utilized under permission, and data analysis was performed at the Taiwan Health and Welfare Data Science Center. The availability of these data is restricted, and they are used under a license specifically for this study. Further details on utilizing the database can be found at https://dep.mohw.gov.tw/DOS/np-2497-113.html (accessed on January 12, 2024).

## CRediT author statement

**Ching-Yu Lee:** Conceptualization, Data curation, Funding acquisition, Investigation, Writing – original draft. **Fung-Chang Sung:** Conceptualization, Funding acquisition, Resources, Software, Visualization, Writing – review & editing. **Peir-Haur Hung:** Conceptualization, Validation, Visualization. **Chih-Hsin Muo:** Formal analysis, Methodology, Resources, Software, Validation. **Meng-Huang Wu:** Conceptualization. **Tsung-Jen Huang:** Conceptualization. **Chih-Ching Yeh:** Data curation, Formal analysis, Investigation, Methodology, Project administration, Resources, Supervision, Visualization, Writing – review & editing.

## Conflicts of interest

The authors declare no competing interests.
